# Malaria in 2022: Challenges and Progress

**DOI:** 10.4269/ajtmh.22-0128

**Published:** 2022-04-12

**Authors:** Philip J. Rosenthal

**Affiliations:** Department of Medicine, University of California, San Francisco, California

Despite the challenges of the pandemic, we have recently seen a number of important advances in malaria. Not long ago, the main malaria story was about improvements, with decreasing worldwide incidence and declarations of malaria elimination in one country after another. Progress continues, but primarily in countries with relatively little malaria. Unfortunately, in many countries hardest hit by malaria, especially in Africa, there has been backsliding, with more cases estimated in 2020 than in 2015. Of great concern, resistance to artemisinins has gained a foothold in Africa. On a brighter note, the first malaria vaccine has been approved by the WHO, and other improved tools to control malaria are available.

## CASES ARE UP; DEATHS ARE WAY UP

As was well-described previously, the incidence of malaria decreased steadily from about 2000 to 2015, with stalling of progress since that time. By the latest WHO estimates (which, notably, include changes in estimates for past years), there were 241 million cases of malaria in 2020—an increase of 6% from 227 million in 2019.^1^ Estimates of malaria deaths included a change in the distribution of mortality in young children, markedly raising estimates for past years. With this new baseline, deaths attributed to malaria increased to 627,000 in 2020, compared with 558,000 in 2019, 562,000 in 2015, and 896,000 in 2000. For 2020, it is estimated that 47,000 of the 69,000 increased deaths, compared with those in the prior year, were a result of service disruptions related to the COVID-19 pandemic.^1^ So, progress against malaria is stalled, and the problem has recently been exacerbated by the challenges of the pandemic.

## INCREASINGLY, THE PROBLEM IS CENTERED IN AFRICA

The malaria problem has been greatest in Africa through recent times, but the imbalance between Africa and the rest of the world has been growing. Case incidence decreased in 2020 in all WHO regions outside Africa. Many countries outside Africa have seen remarkable malaria gains in recent years, with many moving toward elimination. Remarkably, China was certified malaria free by the WHO in 2021. Meanwhile, sub-Saharan Africa accounted for ∼95% of the malaria burden in 2020. Six countries, all in Africa (Nigeria, Democratic Republic of the Congo, Uganda, Mozambique, Angola, and Burkina Faso), accounted for 55% of cases. This burden is overwhelmingly from *Plasmodium falciparum*; *Plasmodium vivax*, which is little seen in most of Africa, now makes up only ∼2% of total global cases. More than ever, although the problem persists in a large part of the tropics, malaria can be appreciated as primarily an African problem.

## ARTEMISININ RESISTANCE IN AFRICA

Artemisinin-based combination therapy (ACT) became the standard of care to treat *falciparum* malaria around the world by soon after the turn of the past century. Within the next decade, resistance to artemisinins, manifest clinically as delayed clearance after treatment with an artemisinin or in vitro as persistence of cultured parasites after brief incubation with dihydroartemisinin, emerged in the Greater Mekong Subregion of Southeast Asia.^2^ This problem has been followed by development of resistance to ACT partner drugs—in particular, piperaquine and mefloquine—accompanied by high rates of ACT treatment failure.^3^^,^^4^ However, the problem of resistance in Southeast Asia has been tempered by the rather low burden of malaria in this region. Of great concern has been the risk of emergence of artemisinin resistance in Africa. Now, this eventuality has been realized. Recent studies showed emergence of mutations in the *P. falciparum* kelch (K13) protein, previously associated with resistance in Southeast Asia, in Rwanda and Uganda,^5^^,^^6^ followed by demonstration of association of these mutations with delayed parasite clearance after artemisinin therapy in both countries.^7^^,^^8^ Indeed, there appear to have been a number of independent emergences of these resistant parasites, suggesting that additional emergences of artemisinin resistance can be expected in Africa. These results are very worrisome, as novel drugs to treat malaria are not expected soon, although it is not yet clear whether ACTs are routinely failing to treat malaria in Africa. For now, improved surveillance for drug resistance is needed across Africa, and strategies under consideration to circumvent resistance include rotation of different ACTs or use of triple ACTs^9^ (containing two partner drugs) to thwart the spread or emergence of resistance to both artemisinins and key partner drugs.

## WHO APPROVAL OF THE RTS,S VACCINE

Extensive efforts to develop malaria vaccines have been going on for decades, and now one program has borne fruit, with WHO endorsement of the RTS,S vaccine, which contains antigenic portions of the circumsporozoite protein (CSP), the hepatitis B surface antigen, and a novel adjuvant. In October 2021 the WHO recommended that the RTS,S/AS01 malaria vaccine be used for the prevention of *P. falciparum* malaria in children living in regions with moderate to high transmission. This was a groundbreaking announcement, with RTS,S becoming the first vaccine approved to prevent a parasitic infection. However, the efficacy of the RTS,S vaccine is modest, with ∼30% to 50% decreases in symptomatic and severe malaria in most trials, and waning protection probably necessitating repeated booster doses. Furthermore, the demands of producing millions of doses of this vaccine are such that quantities necessary to treat all children at risk are some years away. Nonetheless, agreement between developers and funders to produce large quantities of the vaccine later this decade are good news. Also promising are new data on the R21 vaccine, which is also based on CSP antigens, with preventive efficacy ∼75% in African children, considerably greater than that seen in RTS,S trials.^10^

## CAN WE COMBINE A VACCINE WITH DRUGS TO PREVENT MALARIA?

A challenge of the RTS,S vaccine is that it elicits fairly short-lived protection. With this in mind, researchers have explored using the vaccine for short-term protection during the transmission season in parts of Africa with highly seasonal malaria. In a study in Burkina Faso and Mali, RTS,S was studied in combination with the standard in those countries for malaria prevention: seasonal malaria chemoprevention (SMC) with monthly sulfadoxine/pyrimethamine–amodiaquine during the transmission season.^11^ Children who received the vaccine plus SMC experienced fewer episodes of malaria than those randomized only to SMC or only to vaccination. This new strategy of seasonal malaria vaccination offers an interesting means of combining the benefits of each intervention, in particular in areas with highly seasonal malaria. In a very different strategy combining a vaccine with antimalarial drugs, vaccination with purified *P. falciparum* sporozoites with co-administered antimalarials to prevent bloodstream infection induced durable protection against a *P. falciparum* challenge.^12^ Immunization with sporozoites thus offers a validated alternative vaccination strategy, although it entails significant operational challenges.

## MONOCLONAL ANTIBODIES TO TREAT OR PREVENT MALARIA

Major advances have yielded human monoclonal antibodies modified to extend plasma half-life. One such antibody, directed against a portion of CSP, prevented infection in volunteers exposed to infectious mosquitoes.^13^ This work highlights a novel means of preventing malaria. Challenges remain in administering monoclonal antibodies to large numbers of individuals at risk of malaria; but, with resistance to both drugs and insecticides challenging traditional approaches, long-acting antibodies may become an important new weapon against malaria.

## IMPROVED BED NETS

Long-lasting insecticide-treated bed nets (LLINs) have been a key tool to prevent malaria, but their effectiveness has been challenged by resistance of anopheline vectors to pyrethroids, until recently the only compounds incorporated into LLINs. Now, new LLINs incorporating combinations of compounds to control malaria vectors are available. These include LLINs containing a pyrethroid plus 1) the synergist piperonyl butoxide, which inhibits metabolism of pyrethroids by mosquitoes^14^^,^^15^; 2) the insect growth regulator pyriproxyfen^16^; or 3) a second insecticide, chlorfenapyr.^17^ The first two of these new combinations have shown superior malaria-preventing efficacy compared with traditional LLINs; the third shows promise and is now under study. Insecticide resistance and its potential impacts on LLINs and indoor residual spraying programs remain a major concern, but new LLIN products offer improved effectiveness for a well-proved means of malaria control.

## CONCLUSIONS

We are not as close to worldwide malaria eradication as some had predicted a few years ago. Challenges remain, particularly in Africa, and disruptions related to the COVID-19 pandemic have set back progress. However, research on malaria remains very active, leading to important new tools to augment control in the hardest hit areas and to move us toward elimination in many countries. With the current pandemic, there is increased worldwide attention on the control of infectious diseases. We can hope that this attention will spur improved efforts to control and eliminate malaria in the coming years.
